# Supplementary figures and images for: Lymphatic endothelial-cell expressed ACKR3 is dispensable for postnatal lymphangiogenesis and lymphatic drainage function in mice
Source: PLoS One. 2021 Apr 15;16(4):e0249068. doi: 10.1371/journal.pone.0249068 (PMC8049313; doi:10.1371/journal.pone.0249068)

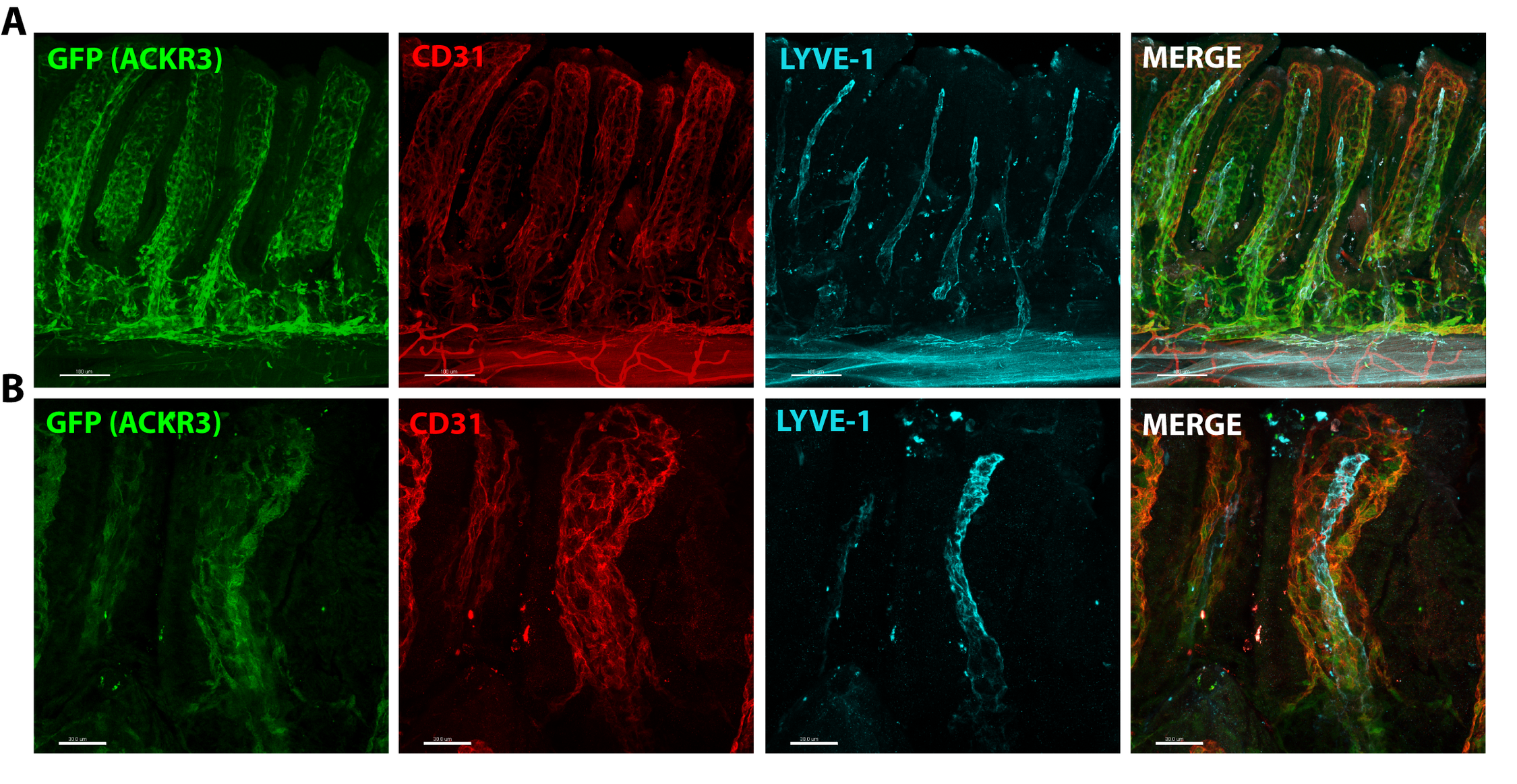

Supplement: S1 Fig — (A) GFP was highly expressed in LYVE-1- CD31+ blood vessels but not detected in LYVE-1+ CD31+ lacteals in villi of the small intestine. Scale bar: 100μm. (B) Higher magnification of a single villi in the duodenum. Scale bar: 30μm. Representative images of one experiment with n = 3 animals. (TIF) [file pone.0249068.s001.tif]

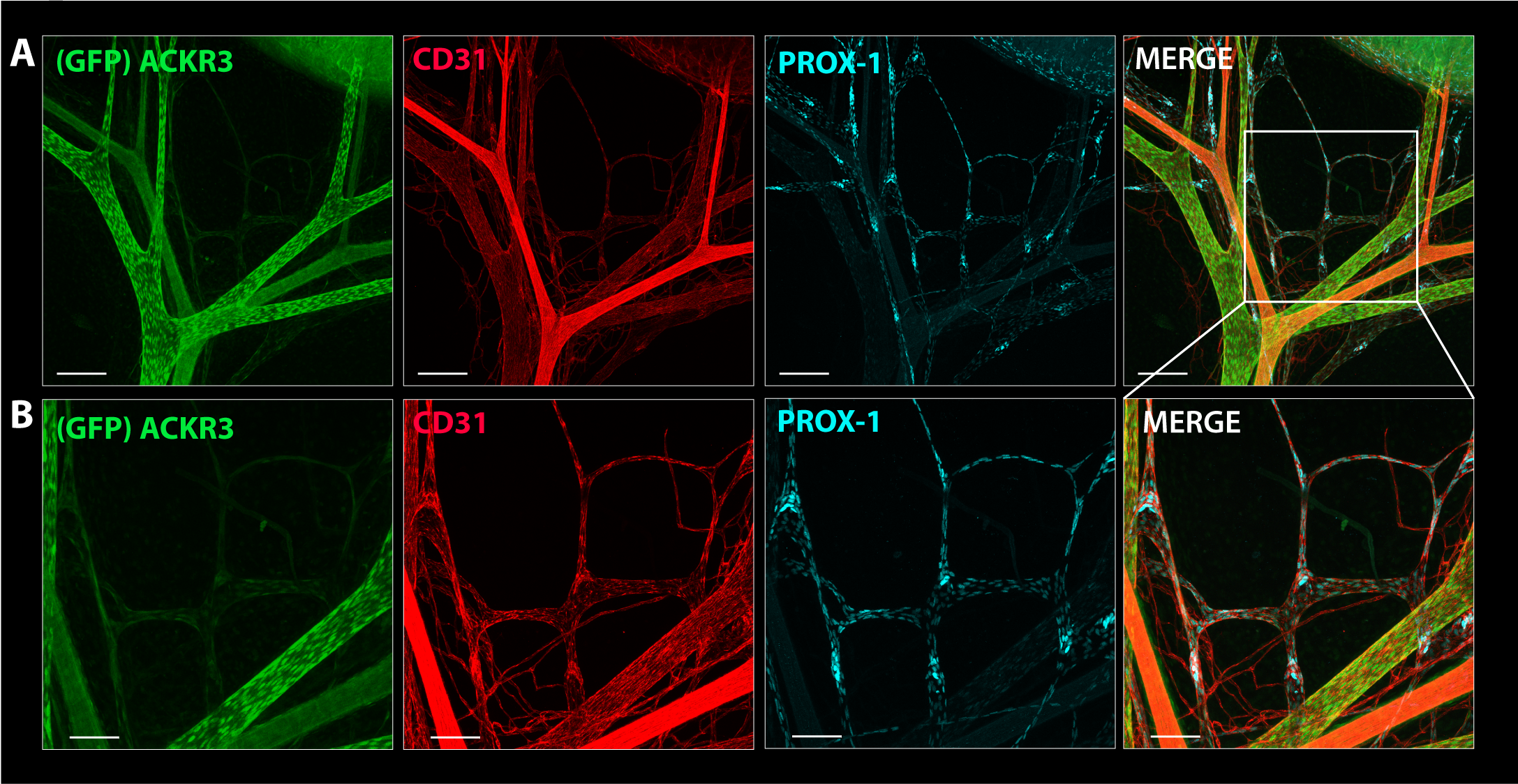

Supplement: S2 Fig — (A) Overview picture of large blood vessels and PROX-1+ lymphatic collectors. Scale bar: 200μm (B) Higher magnification shows GFP expression in lymphatic collectors. Scale bar: 100μm. Representative images of one experiment with n = 4 animals. (TIF) [file pone.0249068.s002.tif]

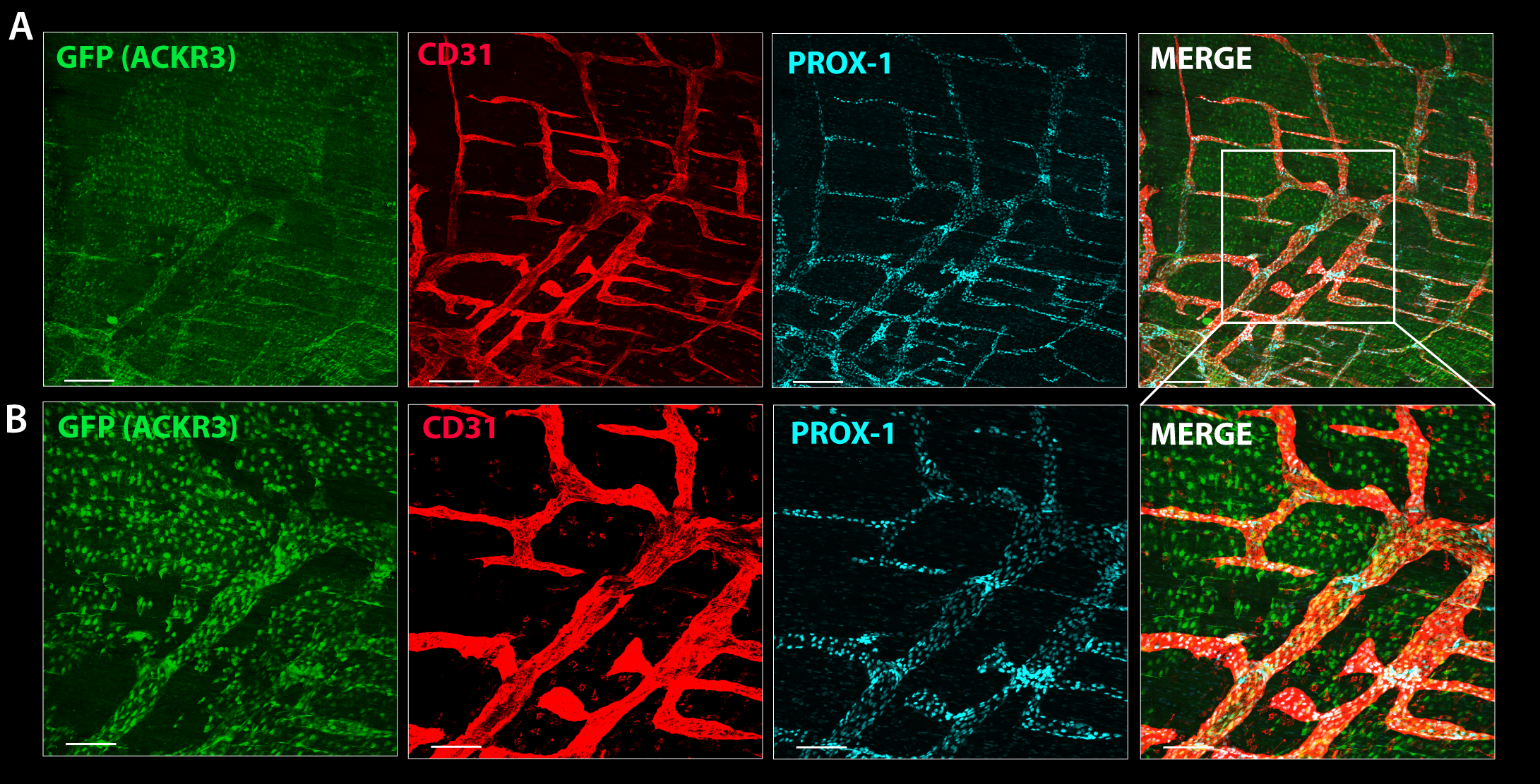

Supplement: S3 Fig — (A) Overview picture of the diaphragmic muscle in an area with high GFP expression in PROX-1+ lymphatic vessels. Scale bar: 200μm (B) Higher magnification. Scale bar: 100μm. Representative images of one experiment with n = 4 animals. (TIF) [file pone.0249068.s003.tif]

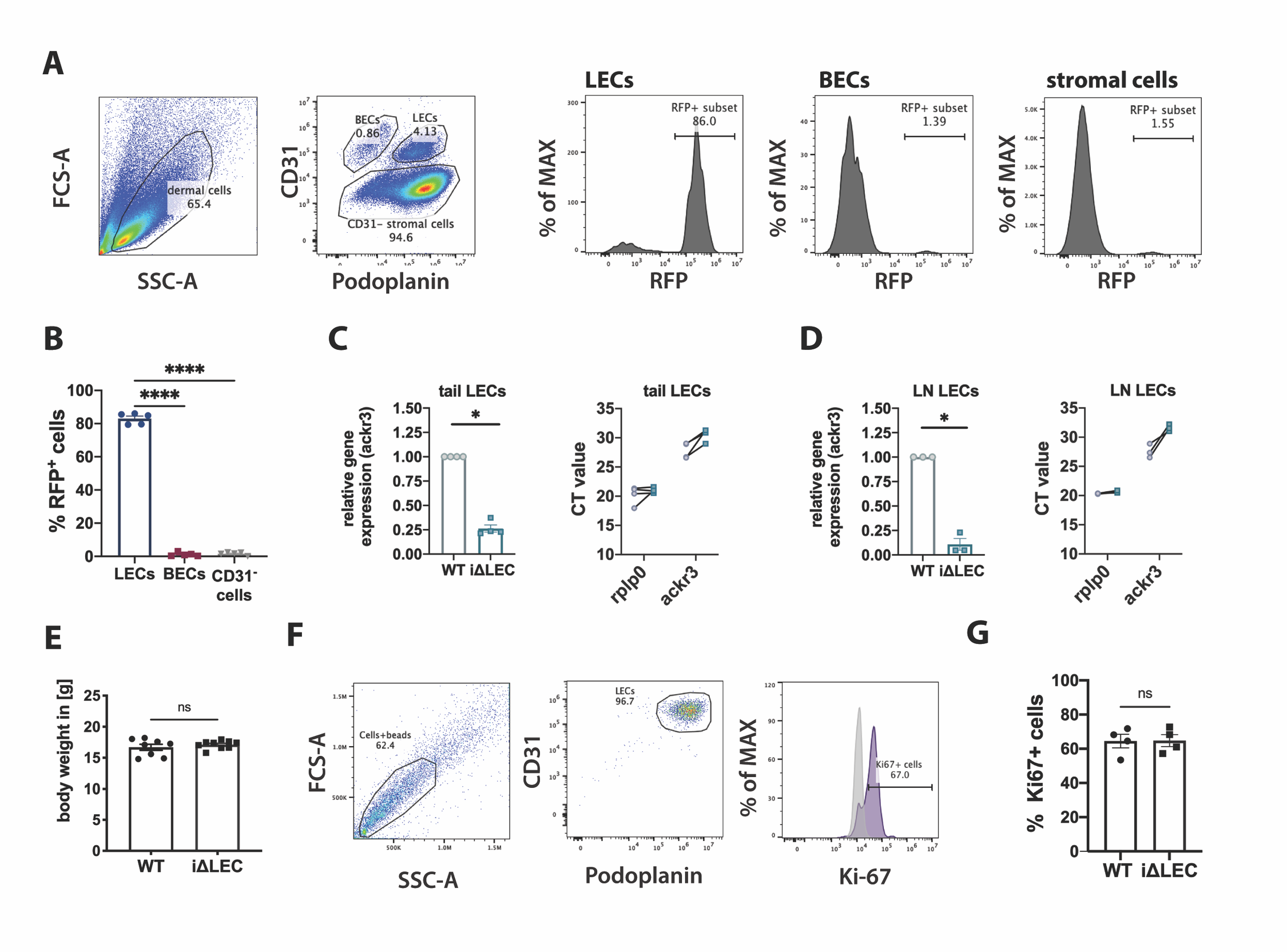

Supplement: S4 Fig — (A) Gating strategy used for the detection of RFP+ LECs in dermal stromal cell cultures isolated from ears of adult tamoxifen-treated animals. (B) % of RFP+ LECs in comparison to the % of RFP+ BECs and CD31- stromal cells, one-way ANOVA, n = 5 animals. (C) Relative ackr3 mRNA expression in isolated dermal tail LECs, n = 4 animals. (D) Relative ackr3 mRNA expression in isolated LN LECs, n = 3 animals. (E) Average body weight of 7-week-old female ACKR3iΔLEC compared to ACKR3WT control animals, Student`s t-test, n = 8 animals. (F) Gating strategy for the detection of the proliferation marker Ki-67 in isolated dermal tail LECs. (G) Proliferation was unaffected in dermal tail skin LECs isolated from ACKR3iΔLEC mice compared to ACKR3WT controls, n = 4 experiments, each involving LECs isolated from one ACKR3iΔLEC and one ACKR3WT mouse. All data in (C- D, G) were analyzed using a Mann- Whitney U- test. (TIF) [file pone.0249068.s004.tif]

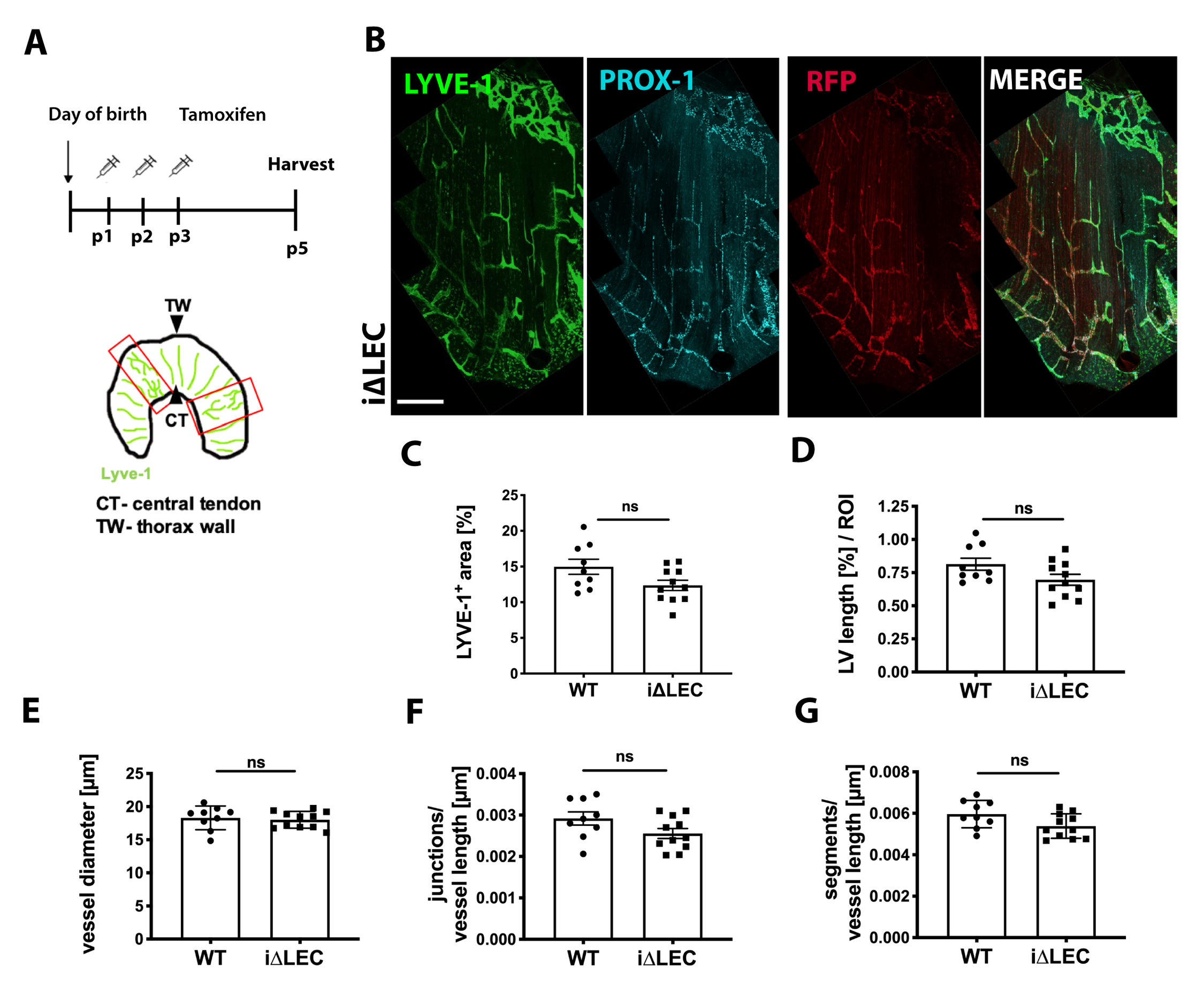

Supplement: S5 Fig — (A) Lymphangiogenesis in the diaphragm was analyzed at p5 following treatment with tamoxifen at p1-p3 in two vessel segments per pup (red frames). (B) Robust RFP expression in lymphatics was detected in ACKR3iΔLEC animals at p5. Scale bar: 300μm. (C) Image based-morphometric analysis of the LYVE-1+ vessel area, (D) total vessel length in %, (E) vessel diameter, (F) number of junctions/branch points per vessel length and (G) the number of segments per vessel length showed no significant difference between ACKR3iΔLEC and ACKR3WT animals. Each data point derives from one pup (n = 9–11) and represents an averaged value of 2–3 quantified diaphragmic images. Student’s t- test. (TIF) [file pone.0249068.s005.tif]

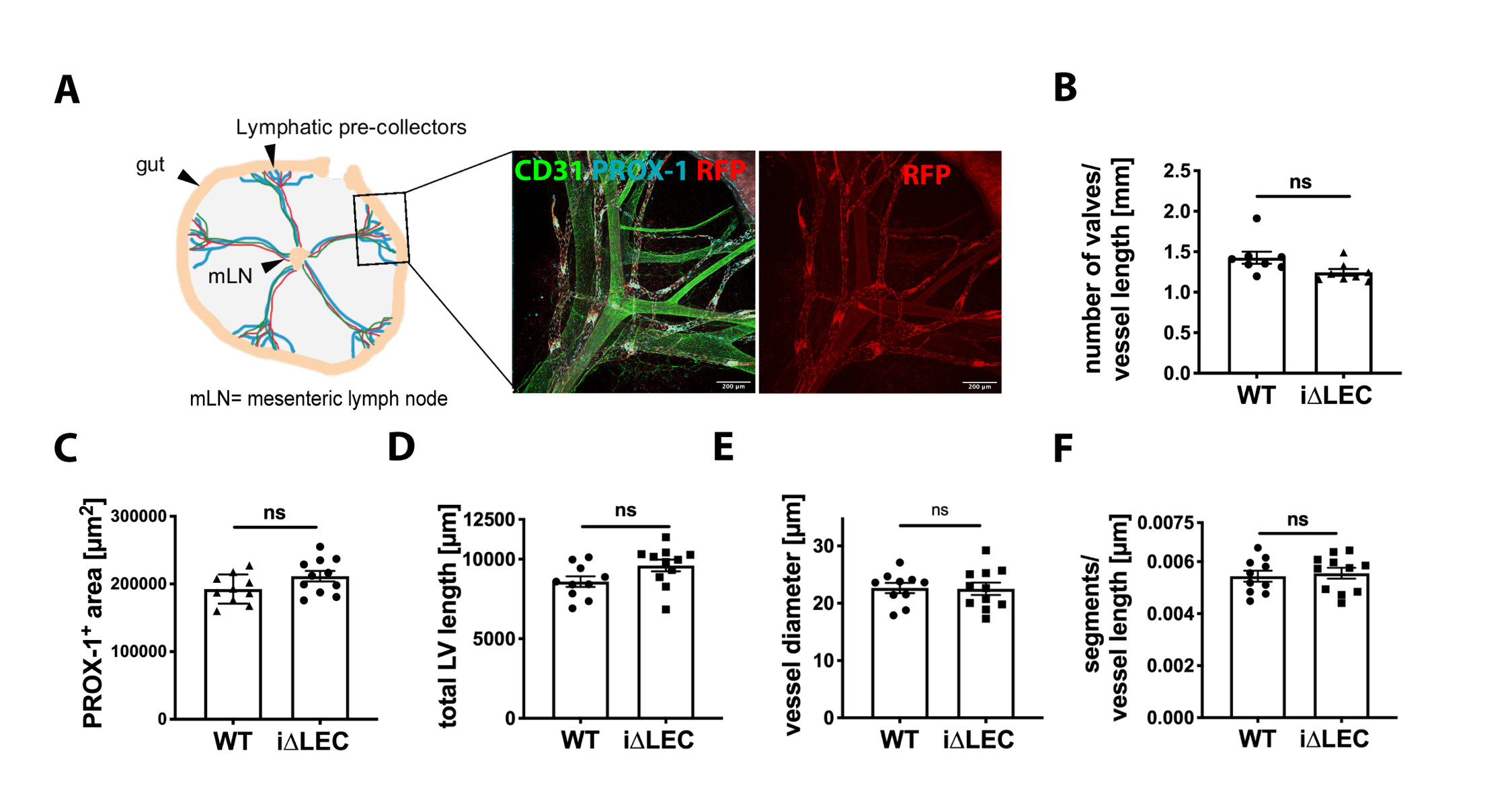

Supplement: S6 Fig — (A) Analyzed area of the mesenteric lymphatic network and RFP reporter expression in PROX-1+ lymphatic vessels at p5 after tamoxifen treatment at p1-p3. Scale bar: 200μm. (B) Comparison of the number of valves/vessel length between ACKR3iΔLEC and ACKR3WT animals. (C) Absolute PROX-1+ area in [μm2]. (D) Total lymphatic vessel length in [μm]. (E) vessel diameter in [μm], (F) Number of segments per total vessel length. Data from three independent experiments are shown as mean ±SEM. Each data point represents an averaged value of 3–5 quantified images of one pup. Student’s t test. (TIF) [file pone.0249068.s006.tif]

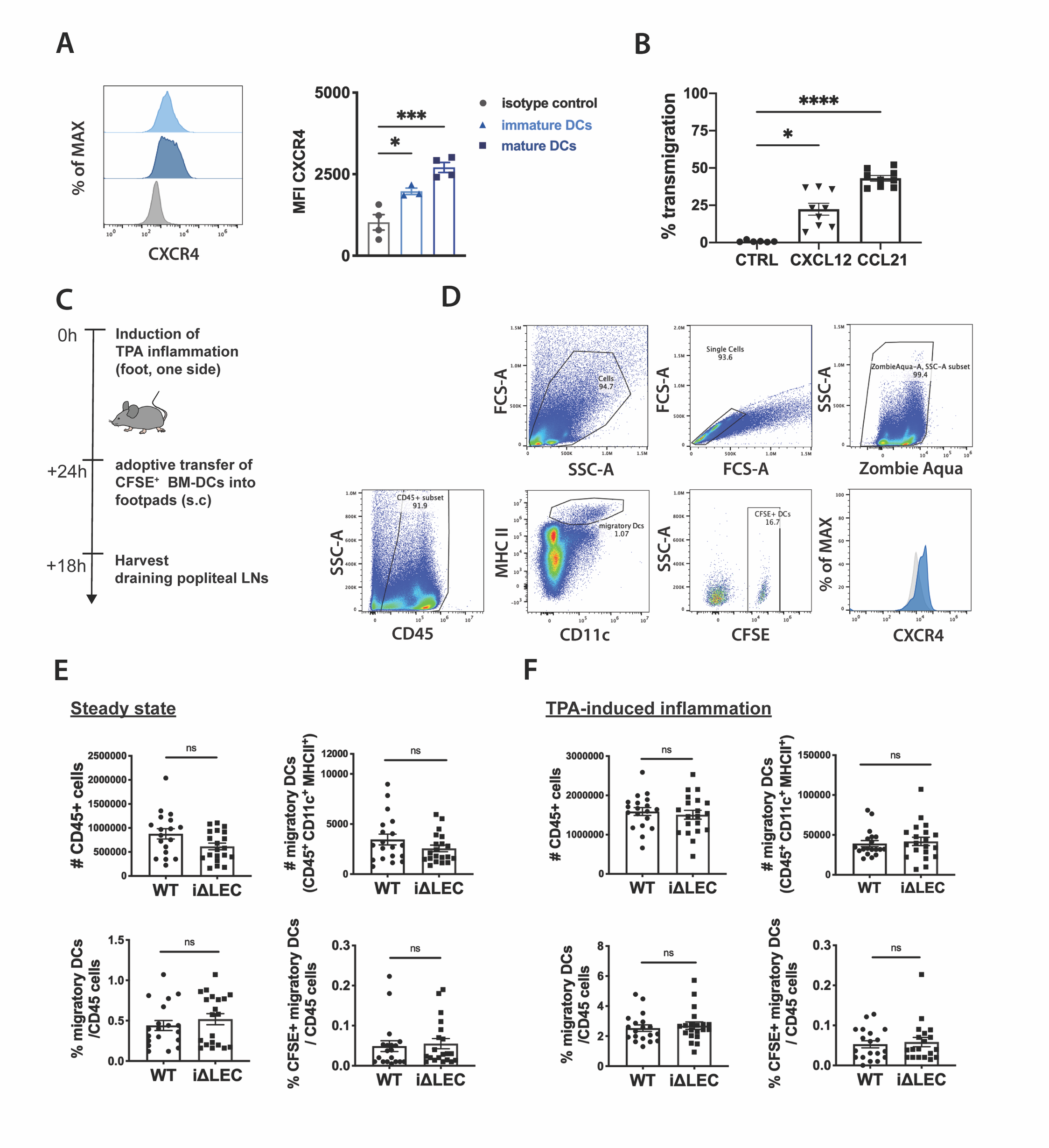

Supplement: S7 Fig — (A) BM-DCs and LPS matured BM-DCs express CXCR4, n = 4 independent experiments, one-way ANOVA (B) CXCL12-mediates transmigration of BM-DCs through monolayers of immortalized LECs, n = 3 independent experiments. Kruskal-Wallis test. (C) CFSE+ BM-DCs were adoptively transferred into steady-state and TPA-inflamed footpads of ACKR3WT and ACKR3iΔLEC animals. Draining popliteal LNs were harvested after 18 h and single cell suspension stained for CD45, CD11c, MHCII and analyzed by FACS. (D) Gating scheme. (E, F) The following quantifications in LN draining (E) steady-state or (F) TPA-inflamed footpads are shown: Total numbers of CD45+ cells and total number of migratory CFSE+ and CFSE migratory DCs (CD45+CD11c+MHCII+), percentage of migratory DC amongst CD45+ cells and percentage of migrated, adoptively transferred DCs (CD45+CD11c+MHCII+CFSE+) amongst CD45+ cells. Pooled data of three independent experiments, each dot represents the value measured in one animal, Mann- Whitney U-test. (TIF) [file pone.0249068.s007.tif]
