# Supplementary material for: Lymphatic endothelial-cell expressed ACKR3 is dispensable for postnatal lymphangiogenesis and lymphatic drainage function in mice
Source: PLoS One. 2021 Apr 15;16(4):e0249068. doi: 10.1371/journal.pone.0249068 (PMC8049313; doi:10.1371/journal.pone.0249068)
Supplement: S1 Methods — (DOCX) [file pone.0249068.s008.docx]

**Supplementary Methods**

**Wholemount immunostaining of intestinal lacteals**

The intestine was harvested after cardiac perfusion from *ACKR3^GFP+/-^* and *ACKR3^GFP+/+^* animals. The small intestine was cut into segments less than 3 cm in length to separate duodenum, jejunum, and ileum. Ileal and duodenal segments were cut open longitudinally along the mesenteric border to expose the lumen. After washing with PBS, intestinal segments were transferred to a 6-cm silicone plate and anchored in the silicon with the luminal side facing up. The tissue was fixed with 2 % fresh PFA/PBS (Sigma-Aldrich) for 2h at 4°C and blocked with Immunomix for 2h at room temperature (RT). Samples were incubated with primary antibodies rabbit anti-mouse Lyve-1 (Angiobio) and rat anti-mouse CD31 (BD Pharmingen), diluted in Immunomix, overnight at 4°C. Then samples were washed with 0.3% Triton-X/PBS at 4°C, and incubated with donkey anti-rat AF594 (Invitrogen) and goat anti-rabbit AF647 (Invitrogen) in 0.3% Triton-X-100/PBS for 2h at 4°C. Slips of single-rowed intestinal villi of the duodenum and the illeum were cut with a razor blade and mounted in Mowiol on glass slides.

**Diaphragm and mesentery**

Pups were sacrificed and diaphragm and intestine were collected and pinned down

into silica-coated wells on cold PBS. For staining of the mesentery lymphatic network, the intestine was pinned in a circle, starting at the side that had been connected to the stomach, in order to span and expose the mesentery for immunostaining. Harvested diaphragms and mesenteries were fixed in 4% PFA/PBS for 2 h at 4°C and then washed with PBS for 45min. Afterwards, tissues were blocked with Immunomix for at least 2 hours at RT. Staining with primary antibodies diluted in Immunomix was carried out overnight at 4°C. Following primary antibodies were used for diaphragms: goat anti-PROX-1 (R&D) and rabbit anti-mouse Lyve-1 (Angiobio). Mesenteries were stained with goat anti-Prox-1 (R&D) and rat anti-mouse CD31 (BD Pharmingen). The following day, samples were washed for 2h with 0.3% Triton-X/PBS before staining with Alexa-conjugated secondary antibodies (Invitrogen) diluted in 0.3% Triton-X/PBS for 2h. Then the samples were washed with PBS for 2h before mounting. Diaphragms were mounted by orienting the pleural side downwards in Mowiol, while intestines were mounted with Vectashield antifade mounting medium (Vector Laboratories) in a chambered borosilicate coverglass system (Nunc).

**Image analysis and processing (diaphragm and mesenteric lymphatic network)**

All confocal images represent maximum intensity projections of z-stacks of single tile or multiple tile scan images that were acquired using confocal z-stacks on a LSM 880 (Carl Zeiss) confocal microscope using Zen Software 2.3 (Carl Zeiss, Version 13.0.0.518). Images were processed with the Image Analysis Software IMARIS (Oxford Instruments, UK, Version 7.6.5). The diaphragmatic muscle was imaged in the costal muscle domain on the pleural side by taking partially overlapping confocal images from the thorax wall to the central tendon (CT) domain (using an EC Plan-Neofluar 10x/0.3 objective from Zeiss), as previously described (1). Per mouse, two full segments of the lymphatic network in the diaphragmatic muscle were analysed. Confocal microscopy images of the diaphragms were eventually automatically merged with Photoshop CS6 (Adobe, San José, CA, USA, Version 13.0.5) and images of both tissues were analyzed using ImageJ (NIH, Bethesda, MD, USA, Version 2.0.0), as previously described (Ochsenbein et al. 2016). Mesenteric lymphatic vessels were imaged in the area, where pre-collecting lymphatic vessels emerge from the intestine. Images were acquired in three to five different areas of the mesentery for analysis. The vessel diameter was calculated by dividing the absolute PROX-1 positive area by the total length of the vessels. The number of valves/total vessel length [µm] was determined using the manual counting feature in ImageJ (NIH).

**Assessment of proliferation and ACKR3 knockdown in isolated primary dermal tail LECs**

Murine primary dermal tail LECs were isolated, similarly as previously described (36), from 6-week-old ACKR3*^i^*^ΔLEC^ and ACKR3^WT^ (WT) littermate control mice, that were injected with tamoxifen after birth. Briefly, murine tails were cut at the base and transferred in HBSS (Invitrogen) supplemented with 1% Pen/Strep (Invitrogen). The skin was removed from the tail and cut into 30mm long skin fragments, which were digested in 1.1 U/mL Dispase II (Roche), for 1h at 37°C with gentle agitation to separate the epidermis from the dermis. Afterwards, dermal fragments were transferred into a new tube and digested with 1mg/mL Collagenase A (Roche) 1,25 mM CaCl_2_ (Sigma-Aldrich) in 20ml PBS for 75 min at 37 °C on a shaker. Digested fragments were then filtered through a 70µM cell strainer, and seeded into two 0.5% gelatin- coated wells of a 6 well cell culture plate in DMEM (Sigma-Aldrich), supplemented with 0.1% Bovine Brain Extract (BBE, Lonza), 10% FBS, 50 μg/mL Heparin (PromoCell), 1% NEAA (Gibco), 1% Pen/Strep and 1% Glutamine (Thermo Fisher Scientific). After 2 days, cells were detached using Accutase® (Sigma-Aldrich) and were incubated for 45min with anti-CD31 antibody-coupled dynabeads (Cellutron Life Technologies and BD Pharmingen Cat, Clone MEC13.3). After purification, Dynabead-coupled cells were washed twice with DMEM and seeded into two wells of a 6 well plate. After reaching confluency, at day 5 or 6 post isolation, CD31+ Dynabead- coupled cells were purified once more, and cells of one well were transferred into 1ml Trizol™ solution (Invitrogen). RNA was isolated and stored, according to the manufacturers protocol. Cells of the second well were used to assess proliferation. To this end, 40 000 cells/well were seeded into 4 wells (technical replicates) of a 24 well plate and Ki67 expression was measured 24h later by flow cytometry. To analyze Ki67 expression, cells were stained with rat anti-mouse CD31-APC, syrian hamster anti-mouse Podoplanin-PE followed by an intracellular staining with anti- Ki67 eFluor 450 (SolA15, ebioscience) using the Foxp3 / transcription factor staining buffer set (ebioscience). Subsequently, cells were acquired on a Cytoflex S apparatus (Beckman Coulter, Brea, CA, USA). Data were analyzed using FlowJo software 10.4.0 (Treestar).

For the assessment of *ackr3* deletion, RNA isolated from tail LECs (and LN LECs, see below) was reverse transcribed using the High Capacity cDNA kit (Thermo Fisher Scientific) and qRT-PCR was performed using the Power Up™ SYBR Green Mastermix (Life Technologies) on a QuantStudio 7 Flex Real-Time PCR System (Applied Biosystems). For the determination of ackr3 deletion, primers were specifically designed in such way, that they are complementary to the transcript at the loxp targeted exon of ACKR3, containing the coding sequence. **Primer sequences:** **mRPLP0** (FW): 5'-AGATTCGGGATATGCTGTTGGC-3', **mRPLP0** (RV): 5'-TCGGGTCCTAGACCAGTCTTC-3', **mACKR3** (FW): 5'-TAAGACCACAGGCTACGACA-3', **mACKR3** (RV): 5'-TGAGGTGTGTGATCTTGCATG-3', **mPROX-1** (FW): 5'-TTTTACACCCGCTACCCCAG-3', **mPROX-1** (RV): 5'-TGGAACCTCAAAGTCATTTGCT-3', **mPDPN** (FW): 5'-AGAGAACACGAGAGTACAACCA-3', **mPDPN** (RV): 5' CGTTTCATCCCCTGCATTATCT-3'.

**Isolation of lymph node LECs (LN LECs)**

LN LECs were isolated as described before (2). In brief, LNs (popliteal, inguinal, axillary, brachial and auricular) were harvested from ACKR3*^i^*^ΔLEC^ and ACKR3^WT^ control mice. After placing LNs into digestion solution, containing 0.25 mg/mL Liberase TL (Roche) and 200 U/mL DNase I (Sigma-Aldrich) in RPMI (Gibco), the LN capsule was broken with a fine gauge needle. Afterwards, LNs were digested for 1h at 37°C and 5% CO_2_. During incubation, the samples were agitated every 15 min by pipetting up and down. After 1h, the LN suspension was passed through a 70μm cell strainer (Invitrogen) and plated into collagen type I- and fibronectin- (PureCol, Advanced BioMatrix /Sigma-Aldrich) (10µg/ml, each) coated cell culture plates in Minimal Essential Medium (MEM)-alpha medium, supplemented with 10% FBS and 1% Penicillin/Streptomycin (P/S) (all from Gibco). Subsequently, cells were cultured at 37°C and 5% CO2 and the medium was changed 24 and 72h after digestion. When reaching confluency, cells were detached with Accutase® (Sigma-Aldrich) and CD31+ endothelial cells were purified, using CD31^+^ microbeads (Miltenyi Biotech). After, the purity was verified by flow cytometry, LN LECs were reseeded into new cell culture plates and cultured for 3-4 days before being lysed in Trizol™ solution (Invitrogen) for RNA isolation (as above).

**Bone marrow derived dendritic cells (BM-DCs)**

BM-DCs were generated from BM extracted from tibia and femurs of mice as described in (3). BM red blood cells were lysed with ACK buffer (4.01 g NH_4_Ac, 0.5 g KHCO_3_, 0.01 g EDTA in 500 ml H_2_O, all from Sigma-Aldrich), and afterwards 5 x 10^6^ cells were plated in 10 ml of DC medium (leukocyte medium + GM-CSF) containing RPMI 1640 (Sigma-Aldrich), 10% FCS, 15 mM HEPES, 1 mM sodium pyruvate, penicillin (100 U/ml), streptomycin (100 μg/ml), L- glutamine (2 mM) (all from Thermo Fisher Scientific), 50 μM β-mercaptoethanol (Sigma-Aldrich) and 80 ng/ml GM-CSF (derived from the supernatant of hypoxanthine-aminopterin-thymidine- sensitive Ag8653 myeloma cells -X63 Ag8.653- transfected with murine GM-CSF cDNA3) into bacterial dishes (Greiner Bio-One). Medium was exchanged on day 3 and 6. On day 9, non- attached cells were harvested and re-seeded into tissue-culture-treated dishes (TPP) in DC medium containing 0.2 μg/ml LPS (Enzo Life Sciences). 24 h later, the supernatant, containing non-attached cells was harvested and DC purity and maturation status were assessed by flow cytometry. Following markers were analyzed: CD11c, MHC-II, CD86, CD80 using PE/Cy7 armenian hamster anti-mouse CD11c, BV421 rat anti-mouse MHC-II, APC rat anti-mouse CD86, FITC armenian hamster anti-mouse CD80 and corresponding isotype controls (all from BioLegend). In a separate staining, LPS matured and immature BM-DCs were also stained for CXCR4 using anti-CXCR4 (clone 2b11, ebioscience) and a respective isotype control.

***In vitro* transmigration assay**

Conditionally immortalized murine lymphatic endothelial cells (imLECs) (4) cultured in media containing 40% DMEM (low glucose), 40% F12-Ham, 20% FBS (all from Gibco), 56 μg/ml heparin (Sigma-Aldrich), 10 μg/ml endothelial cell growth supplement (Sigma-Aldrich), 1% antibiotic antimycotic solution (Fluka), L-glutamine (2 nM; Fluka) were seeded onto the upper side of collagen (Advanced Biomatrix) and fibronectin- (10 μg/ml each) coated- transwell membrane inserts with 5-μm pore size (Corning Life Sciences) and grown to confluence. On the day of the assay, 600µl CXCL12 and CCL21 (100 ng/ml, Peprotech) were added to the bottom well of the transwell, followed by 50 000 BM-DCs, previously labelled with CMFDA (Thermo Fisher Scientific), added into the upper well of the transwell insert. After 4 h, the media in the bottom well was collected and the number of DCs quantified by flow cytometry on a CytoFlex S apparatus (Beckman Coulter).

**Adoptive transfer of bone-marrow derived dendritic cells into footpads of ACKR3^i∆LEC^ and ACKR3^WT^ animals**

BM-DCs were cultured as described above and matured for 12-16h with 0.2µg/ml LPS. For adoptive transfer studies DCs were harvested and labelled with CSFE (5nM) (Thermo Fisher Scientific). Cells were left to rest in a humidified incubator at 37°C and 5% CO_2_ for 30 min in medium after labelling. Subsequently, dead cells were removed by centrifugation over an FBS gradient. Cells were resuspended at 0.75 to 1.5 Mio cells, in 5µl PBS, and injected s.c. into the steady state or TPA- inflamed footpads of ACKR3^i∆LEC^ or WT littermate control mice. TPA-induced inflammation of the footpad was induced 24h prior, by applying 2µg TPA dissolved in acetone to the footpad (of one side, as described above). Popliteal LNs were harvested after 18 to 20 h, passed through a 40 µm cell strainer and blocked for 10min on ice with an anti-CD16/32 Fc-receptor blocking antibody prior to staining with anti-CD11c, anti-MHC II, anti-CD45 (all from Biolegend), anti-CXCR4 (clone 2b11, eBioscience) and Zombie Aqua for detection of DC subsets and CXCR4. Samples were acquired on a Cytoflex S apparatus (Beckman Coulter, Brea, CA, USA) using CytExpert software and analyzed with FlowJo software 10.4.0 (Treestar).

1. Ochsenbein AM, Karaman S, Proulx ST, Goldmann R, Chittazhathu J, Dasargyri A, et al. Regulation of lymphangiogenesis in the diaphragm by macrophages and VEGFR-3 signaling. Angiogenesis. 2016;19(4):513-24.

2. Commerford CD, Dieterich LC, He Y, Hell T, Montoya-Zegarra JA, Noerrelykke SF, et al. Mechanisms of Tumor-Induced Lymphovascular Niche Formation in Draining Lymph Nodes. Cell Rep. 2018;25(13):3554-63 e4.

3. Lutz MB, Kukutsch N, Ogilvie AL, Rossner S, Koch F, Romani N, et al. An advanced culture method for generating large quantities of highly pure dendritic cells from mouse bone marrow. J Immunol Methods. 1999;223(1):77-92.

4. Vigl B, Aebischer D, Nitschke M, Iolyeva M, Rothlin T, Antsiferova O, et al. Tissue inflammation modulates gene expression of lymphatic endothelial cells and dendritic cell migration in a stimulus-dependent manner. Blood. 2011;118(1):205-15.
